# Clinical Society

**Published:** 1897-02-20

**Authors:** 


### Medical Progress and Hospital Clinics.

[*The Editor will be glad to receive offers of co-operation and contributions from members of the profession. All letters should be addressed to* The Editor, at the Office, 28 & 29, Southampton Street, Strand, London, W.C.]

### CLINICAL SOCIETY.

At the last meeting, held on February 12th, Mr. Ernest Lane read a paper on "Two Cases of Gastro-Enterostomy," in one of which Murphy's button had failed to produce pressure atrophy, owing apparently to a deficient amount of strength in the spring. This led to some discussion in regard to the comparative advantages of simple sewing and the use of mechanical contrivances.

One speaker drew attention to the occurrence of vomiting after the operation, and raised the question whether this might not be due to closure of the end of the button by the opposite wall of the jejunum. He had operated by Halstead's method, and had attached the jejunum to the posterior portion of the stomach so as to avoid bagging, and he had found that by so doing the dilatation rapidly went away. He had known two or three cases of leakage after using Murphy's button, and, as to the time required, Halstead's method did not take more than a quarter of an hour.

Mr. Barker said that, although he would not be an advocate of the button, yet he would not attribute the vomiting which had occurred to its presence, for vomiting occurred where no button at all was used. It was probably due to a simple reflux of the duodenal contents into the stomach. In his first case of suture it had been a troublesome complication, which, however, was relieved by placing the patient in the vertical or semi-recumbent position. He thought, then, it was a little unfair to attribute these evils to the apparatus which had been used. His own experience, however, was in favour of ordinary suture without any mechanical contrivances, and he thought the opinion was gaining ground that simple suture was the best.

Mr. Lane, in reply, said that washing out the stomach stopped the vomiting, which confirmed the idea that the cause was that suggested by Mr. Barker.

Mr. Stephen Paget described a remarkable series of cases of voracious hunger and thirst after injury or disease of the brain. Some of these cases were connected with disease in the tempero-sphenoidal lobe, while in those in which the condition followed upon injury it seemed not unlikely that this lobe had been involved, and Mr. Paget suggested the existence of special centres in this part of the brain connected with the sensation of hunger and of thirst.

Some of the cases described were very striking, both in regard to the suddenness with which the symptoms were developed after accidents, and in regard to the enormous quantities of food and drink which were consumed by the patients. One patient drank fifty-two pints in one day, and another drank fifty-four pints the day after the accident. In one case the patient became thirsty directly after the accident, and drank five pints before the first micturition. In regard to hunger there was almost a comic side to the condition, the voracity was in some cases so extreme. The state described was, however, quite distinct from diabetes. There was no sugar in the urine.

Mr. Wallis had had a case of excessive voracity in a boy. He was not very thirsty, but he would eat anything. He died, and the whole of the tempero-sphenoidal lobe was found to be one large abscess cavity, which he thought went against the hypothesis of the irritation of a special centre in that part.

The President said that he could not at the moment recall to mind any striking example of the condition which had been described. Yet he had seen a large number of cases of nerve disease, and it seemed almost impossible to believe that no such cases had come before him, which was but another illustration of the fact that we do not see what we do not look for. Probably some irritation of the gastric branch of the vagus or its central root was involved in these cases; unfortunately we did not know where the cerebral root of the vagus lay. He had seen cases of ravenous appetite in connection with the gastric crises of Charcot, there being pain and vomiting, and when they ceased the patient becoming very voracious. This was no doubt from irritation of the bulbar nucleus of the vagus, and it was conceivable that the same thing might arise from disturbance affecting the cerebral roots.

Dr. E. W. Goodall related two cases of enteric fever fatal during the third and fifth week respectively in which there was no intestinal ulceration.

In the discussion which followed, Dr. Sidney Phillips said that, in addition to the two cases he had reported to the society in 1891, he had seen several cases of typhoid fever without intestinal ulceration. He had quite lately seen a case of fatal typhoid fever with only one ulcer, and that of the smallest size. If such cases could come into the post-mortem room we might fairly infer that in a great many of the cases which recovered there was no ulceration.

This had an important practical bearing on the question of feeding. Dr. Barrs had lately advocated giving typhoid patients a more solid diet, and, if one could be sure that there was no ulceration, many patients would do better with a more liberal diet than they received at the present, but, as it was not possible to be certain, he thought liquid diet should be adhered to.

Dr. Phillips also drew attention to the considerable change in the type of the disease that had occurred during recent years, much less diarrhœa being present, in fact often constipation, and concurrently much less ulceration, being seen in the post-mortem room. Typhoid fever was, in fact, becoming a disease with often very slight abdominal symptoms and much less eruption than formerly, but with a great tendency to relapse.

